# Tuberculosis and its association with CD4^+^ T cell count among adult HIV positive patients in Ethiopian settings: a systematic review and meta-analysis

**DOI:** 10.1186/s12879-020-05040-4

**Published:** 2020-05-07

**Authors:** Demeke Geremew, Mulugeta Melku, Aklilu Endalamaw, Berhanu Woldu, Alebachew Fasil, Markos Negash, Habtamu Wondifraw Baynes, Habtamu Geremew, Takele Teklu, Tekalign Deressa, Belay Tessema, Ulrich Sack

**Affiliations:** 1 Department of Immunology and Molecular Biology, School of Biomedical and Laboratory Sciences, University of Gondar, P.o.Box: 196, Gondar, Ethiopia; 2 Department of Hematology and Immunohematology, School of Biomedical and Laboratory Sciences, University of Gondar, Gondar, Ethiopia; 3 Department of Pediatrics and Child Health Nursing, School of Health Sciences, College of Medicine and Health Sciences, Bahir Dar University, Bahir Dar, Ethiopia; 4 Department of Clinical Chemistry, School of Biomedical and Laboratory Sciences, University of Gondar, Gondar, Ethiopia; 5 Department of Reproductive Health, School of Public Health, Debre Markos University, Debre Markos, Ethiopia; 6 Ethiopian Public Health Institute, HIV/AIDS and TB Research Directorate, Addis Ababa, Ethiopia; 7 Department of Medical Microbiology, School of Biomedical and Laboratory Sciences, University of Gondar, Gondar, Ethiopia; 8 Institute of Clinical Immunology, Medical Faculty, University of Leipzig, Leipzig, Germany

**Keywords:** Tuberculosis, HIV, CD4^+^ T cell, Meta-analysis, Ethiopia

## Abstract

**Background:**

Tuberculosis (TB) and HV have been intertwined and makeup a deadly human syndemic worldwide, especially in developing countries like Ethiopia. Previous studies have reported different TB incidences and its association with CD4^+^ T cell counts among HIV positive patients in Ethiopia. Thus, the goal of this meta-analysis was, first, to determine pooled incident TB among adult HIV positive patients, and second, to assess the association between incident TB and baseline CD4^+^ T cell count strata’s.

**Methods:**

We searched PubMed, Cochrane library, Science Direct and Google scholar databases from June 1 to 30, 2018. The I^2^ statistics and Egger’s regression test was used to determine heterogeneity and publication bias among included studies respectively. A random effects model was used to estimate pooled incident TB and odds ratio with the respective 95% confidence intervals using Stata version 11.0 statistical software.

**Results:**

A total of 403 research articles were identified, and 10 studies were included in the meta-analysis. The pooled incident TB among adult HIV infected patients in Ethiopia was 16.58% (95% CI; 13.25–19.91%). Specifically, TB incidence in Pre-ART and ART was 17.16% (95% CI; 7.95–26.37%) and 16.24% (95% CI; 12.63–19.84%) respectively. Moreover, incident TB among ART receiving patients with baseline CD4^+^ T cell count < and > 200 cells/mm^3^ was 28.86% (95% CI; 18.73–38.98%) and 13.7% (95% CI; 1.41–25.98%) correspondingly. The odds of getting incident TB was 2.88 (95% CI; 1.55–5.35%) for patients with baseline CD4^+^ T cell count < 200 cells/mm^3^ compared to patients with baseline CD4^+^ T cell count > 200 cells/mm^3^.

**Conclusion:**

High incident TB among adult HIV positive patients was estimated, especially in patients with CD4^+^ T cell count < 200 cells/mm^3^. Therefore, Early HIV screening and ART initiation, as well as strict compliance with ART and increasing the coverage of TB preventive therapy to more risky groups are important to prevent the problem.

**Trial registration:**

Study protocol registration: CRD42018090802.

## Background

Tuberculosis (TB) is one of the major problems of mankind worldwide, especially after the emergence of HIV pandemic, TB is resurging mainly in resource limited areas of sub-Saharan Africa [1]. Even with the advancement of TB/HIV control programs, the twin widespread emergence of HIV and TB makes them the deadly syndemic of mankind. Many epidemiological studies showed that HIV is a driving force for TB incidence, and people living with HIV (PLWH) have higher risk of developing incident TB [2, 3]. It has been reported that TB is the most common opportunistic infection among HIV positive patients, and recently 12% of the 9.6 million new TB cases were among HIV infected patients [4]. The risk of getting incident TB in PLWH is 21 times greater than the population without HIV comorbidity [5].

Previous studies clearly demonstrated that HIV causes immunosuppression directly by depletion of host CD4^+^ T lymphocytes. As a result of lymphocytopenia and downregulation of these immune cells, vulnerability to TB diseases is increased in HIV positive patients [6, 7]. Moreover, HIV infected patients with decreased CD4^+^ T cell count is associated with increased risk of TB, especially CD4^+^ T lymphocyte count < 200 cells/mm^3^ is much more accompanied with higher TB incidence [8, 9].

Therefore, CD4^+^ T lymphocyte count remains the best indicator of a patient’s immunological and clinical status, the risk of opportunistic infections like TB, and supports diagnostic decision making, particularly for patients with advanced HIV disease [10]. In Ethiopia, individual studies showed inconsistent TB incidence among adult HIV infected patients ranging from 8.3 [11] to 29.06% [12]. Besides, although CD4^+^ T cell count in PLWH impacts TB risk, the quantitative connection between CD4^+^ T cell count and TB risk is not well documented in our country. Thus, we sought to provide a meta-analysis estimate of pooled incident TB among adult HIV positive patients and its association with baseline CD4^+^ T cell counts for better planning and execution of screening programs, regular follow ups and prevention approaches.

## Methods

### Definitions

We have used the following terms in this meta-analysis; incident TB defined as all new TB cases from the begging to the last day of observation. It was calculated by dividing the number of new TB cases by total number of study participants at risk at the begging of observation, and times by 100. Further, the term “incident TB” and “TB incidence” was used interchangeably throughout this manuscript as it was also evidenced elsewhere [13, 14]. Pooled incident TB described as an estimate considered from reported TB incidences of all included studies. Nevertheless, this proportion does not adjust whether the study cohorts were within 6 or >  6 months of follow up for HIV infected patients on pre-ART.

### Reporting and protocol registration

This review was conducted based on Preferred Reporting Items for Systematic Reviews and Meta-Analyses (PRISMA) statement guideline [15]. This study was registered in International Prospective Register of Systematic Reviews (PROSPERO) database with protocol number, CRD42018090802.

### Search strategy

An inclusive literature search was made using PubMed, Cochrane library, Science Direct and Google scholar databases from June 1 to 30, 2018. The search term used for retrieving records in PubMed on 07/08/2018 was: [“human immunodeficiency virus” OR “HIV” AND “CD4^+^ T cell count” OR “CD4 count” AND (Tuberculosis OR TB) AND (infection OR incidence) AND “Ethiopia”]. The PubMed search string was developed in accordance with the Medical Subject Headings thesaurus, (Additional file 1). Moreover, grey literature was also searched in google and from reference lists of pertinent articles to retrieve additional studies. Endnote version 7 (Thomson Reuters, London) reference manager software was used to remove duplicate entries and manage the citation process.

### Inclusion and exclusion criteria

All original research articles conducted in Ethiopian settings that fulfill the following criteria were included in this meta-analysis. Articles with cohort study design, studies reporting TB incidence among adult HIV positive patients conducted only in Ethiopia, articles with a clear participants’ ART status and incident TB report, and studies published in English were considered in this meta-analysis. However, studies with participants below the age of 15 years were excluded from our analysis due to their differences in TB and HIV natural history from adults.

However, review articles, conference abstracts and proceedings, editorials and case reports, studies without full-text access, and studies that investigated patterns of drug resistance only without incidence were excluded. Likewise, studies that reported prevalence of TB among adult HIV positive patients were also excluded.

### Outcome of interest

The primary outcome of interest was the proportion of incident TB among adult HIV infected patients in Ethiopia. Moreover, the results have been stratified by ART status (Pre-ART versus ART), ART follow up time (6 months versus > 6 months) and baseline CD4^+^ T cell counts (baseline CD4^+^ T cell counts < 200 versus > 200 cells/mm^3^). Secondly, we have also determined the odds ratio of developing incident TB among adult HIV infected patients receiving ART with baseline CD4^+^ T cell counts < 200 versus > 200 cells/mm^3^.

### Data extraction and quality assessment

Two reviewers (DG and AE) independently examined the identified studies for inclusion and extracted relevant data. Disagreement between the two reviewers was resolved by discussion and articles were included after consensus was reached. Moreover, if the discrepancy could not be fixed, a third author (MM) was involved to examine the article and resolve the inconsistency. The following parameters were extracted from each included study: study author and year of publication, study area/region, study design, study period, participants’ ART status and age, laboratory methods employed to diagnose TB whenever reported, sample size, ART follow up time to report incident TB, number of participants with incident TB, number of participants with baseline CD4^+^ T cell count less 200 cells/mm^3^ and with or without incident TB, number of participants with baseline CD4^+^ T cell count > 200 cells/mm^3^ and with or without incident TB.

The quality of included studies was verified by using Joanna Brigg’s Institute (JBI) quality assessment checklist for cohort studies [16] by two independent authors (DG, AE) and a third review author (MM) was also involved whenever necessary. That is, each study data was cleared based on the research design and study population (adult HIV positive patients in Ethiopia on pre-ART or ART), reported data quality (incident TB data, and incident TB report based on baseline CD4^+^ T cell count strata whenever reported), and the correctness of the method employed to diagnose incident TB whenever reported.

### Statistical analysis

Data was extracted using Microsoft Excel, then it was exported into Stata version 11.0 (StataCorp, College Station, TX, USA) for further analysis. The random effects model (DerSimonian-Laird method) [17] was used for pooled incidence analysis and odds ratio (OR) with the respective 95% confidence intervals (95% CIs). Nevertheless, for studies with small or large prevalence, near 0 or 1, the inverse variance method adds disproportionately large weight, variance becomes small, and the calculated CI may lie outside of the 0 to 1 range [18]. Thus, we used Freeman Tukey arcsine methodology to address stabilizing variances [19].

Heterogeneity between included studies was determined using the I^2^ statistics. The I^2^ values of 25, 50 and 75% was considered as low, medium and high heterogeneity, respectively [20]. Although the use of I^2^ in assessing heterogeneity may mislead, studies with relatively large I^2^ may be pooled when the clinical heterogeneity is acceptable [21]. Clinical heterogeneity could be partly patient baseline characteristics but not on the outcome measurement scale, statistical heterogeneity. Egger’s test (a statistical analogue for funnel plot) was used to assess publication bias [22]. The robustness of the pooled estimate and the impact of a single study on aggregate result was figured out by sensitivity analysis. Based on the JBI checklist, all studies involved in this analysis were having a quality score of 50% and above.

## Results

### Identification and documentation of studies

**Fig. 1:**
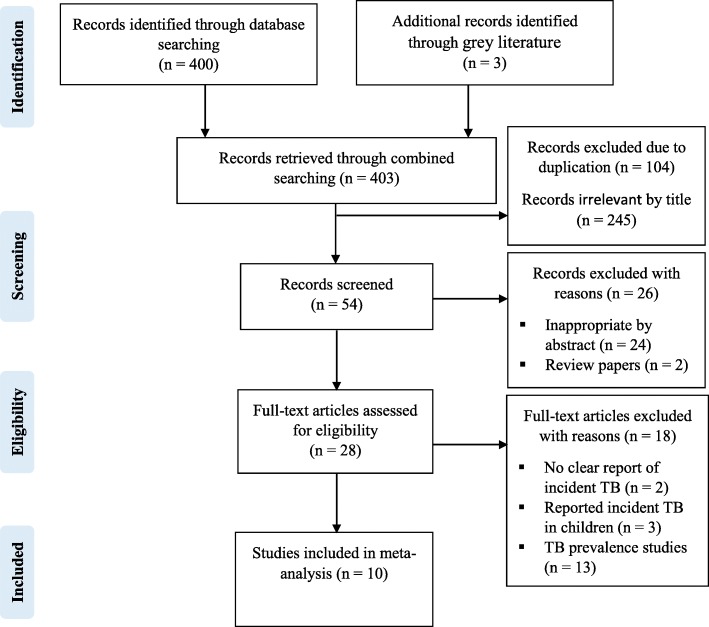
PRISMA flow chart for the studies screened, reviewed and included

The overall literature search yielded a total of 403 potential articles, of which 28 were selected for detailed full text review and 10 studies were found to be appropriate and included in the quantitative analysis (Fig. 1).

### Characteristics of included studies

All the studies included were cohort studies and were published from 2012 to 2017. Among the 10 studies subjected to the meta-analysis, 2 articles [11, 23] reported incident TB on pre-ART, 6 studies on ART [24–29] and 2 studies [12, 30] in both pre-ART and ART receiving HIV positive patients. On the other hand, 2 articles [11, 23] reported incident TB among pre-ART HIV infected patients within 6 months of follow up, whereas 2 studies [12, 30] didn’t indicate the follow up time clearly. In the meantime, 2 articles [24, 25] and 4 studies [26–29] reported incident TB within 6 and above 6 months of ART follow up respectively. Of the 10 studies, 7 articles [11, 12, 23–26, 29] reported incident TB regardless of the baseline CD4^+^ T cell count while 3 studies [27, 28, 30] stated incident TB data on ART in both baseline CD4^+^ T cell count < 200 and > 200 cells/mm^3^.

The age of the participants were ranging from 15 to 64 years old. The studies considered in the meta-analysis have provided incident TB data among a total of 10,074 (1904 on pre-ART and 8170 on ART) HIV infected patients. Of the total 10,074 participants, 1309 (328 on pre-ART and 981 on ART) participants developed incident TB. Moreover, three studies [27, 28, 30] exclusively reported incident TB data on 1290 HIV positive patients on ART based on participants baseline CD4^+^ T cell count status. Namely, there were 188 incident TB cases among HIV infected patients on ART with CD4^+^ T cell count < 200 cells/mm^3^ and 91 incident TB cases with CD4^+^ T cell count > 200 cells/mm^3^ (Table 1).

**Table 1:** Characteristics of included studies (*n* = 10)

Study author	Study region	Study design	Study period	Age (years)	Specimen	Methods employed	ART status	Sample size	TB incidence follow up time	Incident TB (%)	Participants’ with CD4^+^ T cell counts < 200 cells/mm^3^		Participants’ with CD4^+^ T cell counts > 200 cells/mm^3^	
											Events	Total	Events	Total
Balcha et al., 2014 [23]	Oromia	PCS	Oct2011-Mar2013	> 18	Sputum, Urine	Microscopy, Culture, Xpert and LAM	Pre-ART	757	6 months	16.91	–	–	–	–
Assebe et al., 2015 [11]	Oromia	RCS	Jan2008-Feb2012	15–64	Sputum	Microscopy and CXR	Pre-ART	588	6 months	8.33	–	–	–	–
Bekele et al. 2017 [12]	SNNP	RCS	Sep2009-Aug2010	> 15	N/S	N/S	Pre-ART	422	Not clearly stated	31.99	–	–	–	–
							ART	132		19.70	–	–	–	–
Ahmed et al. 2015 [27]	Afar	RCS	July2010-May2015	> 15	N/S	N/S	ART	451	> 6 months	26.39	62	168	57	283
Dalbo et al. 2016 [30]	SNNP	RCS	Dec 2014-Jan2015	> 15	Sputum	Microscopy and culture	Pre-ART	137	Not clearly stated	11.68	–	–	–	–
							ART	359		25.07	61	200	29	159
Abossie et al. 2017 [24]	SNNP	RCS	Sep2010-Aug 2011	25–34	Sputum	Microscopy	ART	271	6 months	18.08	–	–	–	–
Edessa et al. 2014 [25]	Addis Ababa	RCS	Feb2013-May2013	> 18	N/S	N/S	ART	742	6 months	10.78	–	–	–	–
Semu et al. 2017 [26]	Addis Ababa	RCS	July2012-Aug2012	Adult	N/S	N/S	ART	2524	> 6 months	10.97	–	–	–	–
Nigussie et al. 2015 [28]	Addis Ababa	RCS	Aug2014-May2015	> 15	Sputum	Microscopy and CXR	ART	480	> 6 months	14.58	65	323	5	157
Kassa et al. 2012 [29]	Addis Ababa	RCS	2005–2009	> 15	N/S	N/S	ART	3211	> 6 months	8.41	–	–	–	–

**Keys:***ART* Antiretroviral therapy, *CXR* Chest x-ray, *N/S* Not specified, *PCS* Prospective cohort study, *RCS* Retrospective cohort study, *SNNP* Southern nations nationalities and peoples of Ethiopia

### Meta-analysis

#### Heterogeneity and publication bias

**Fig. 2:**
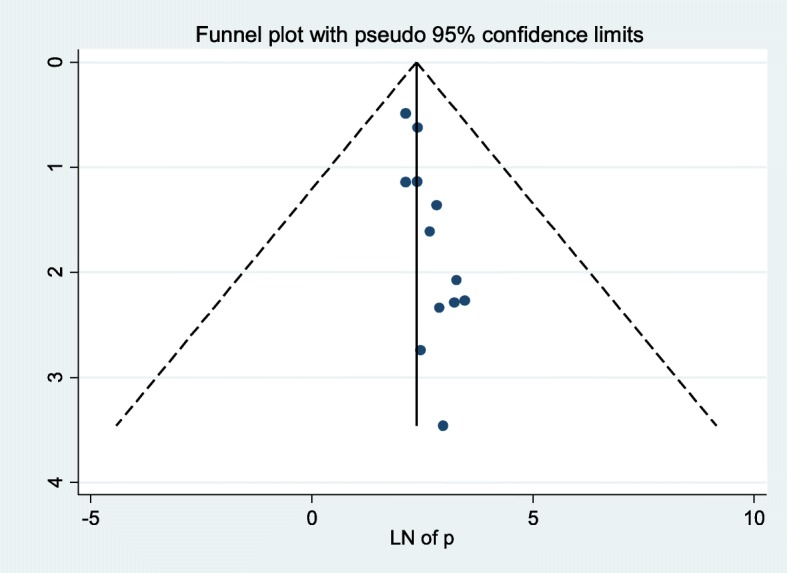
Funnel plot, evaluating the existence of publication bias for the analysis of pooled incident TB

The existence of heterogeneity and publication bias was determined within included studies. Consequently, there was considerable heterogeneity across ten included studies in this meta-analysis (I^2^ = 95.6%). The Egger’s test for publication bias was marginally insignificant (*p* = 0.07), indicating no evidence of publication bias within included studies. Besides, funnel plot was also depicted to illustrate the presence/absence of publication bias (Fig. 2).

#### Sensitivity analysis

Sensitivity analysis showed that the effect of individual studies on pooled estimate was insignificant, suggesting the robustness of aggregated estimate. Therefore, the pooled incident TB among HIV infected patients was steady and reliable when examined by neglecting one study at a time (Table 2).

**Table 2:** Sensitivity analysis of pooled incident TB among adult HIV positive patients on pre-ART and ART in Ethiopia

Study omitted	Estimate	95% CI
Balcha et al. 2014 [23]	16.554401	13.042163, 20.066639
Assebe et al. 2015 [11]	17.416502	13.771809, 21.061195
Bekele et al. 2017^a^ [12]	15.120082	12.209677, 18.030487
Dalbo et al. 2016^a^ [30]	16.986979	13.485174, 20.488785
Ahmed et al. 2015 [27]	15.636593	12.475107, 18.798079
Abossie et al. 2017 [24]	16.450867	12.984303, 19.917431
Edessa et al. 2014 [25]	17.186029	13.488454, 20.883606
Semu et al. 2017 [26]	17.243677	13.042066, 21.445288
Bekele et al. 2017^a^ [12]	16.354092	12.918216, 19.789968
Dalbo et al. 2016^a^ [30]	15.805243	12.532356, 19.078131
Nigussie et al. 2015 [28]	16.78417	13.223361, 20.34498
Kassa et al. 2012 [29]	17.459826	13.627331, 21.29232
Combined	16.580467	13.251581, 19.909352

^a^the author reported incident TB in both pre-ART and ART patients in a single study

#### Pooled incident TB among HIV positive patients

**Fig. 3:**
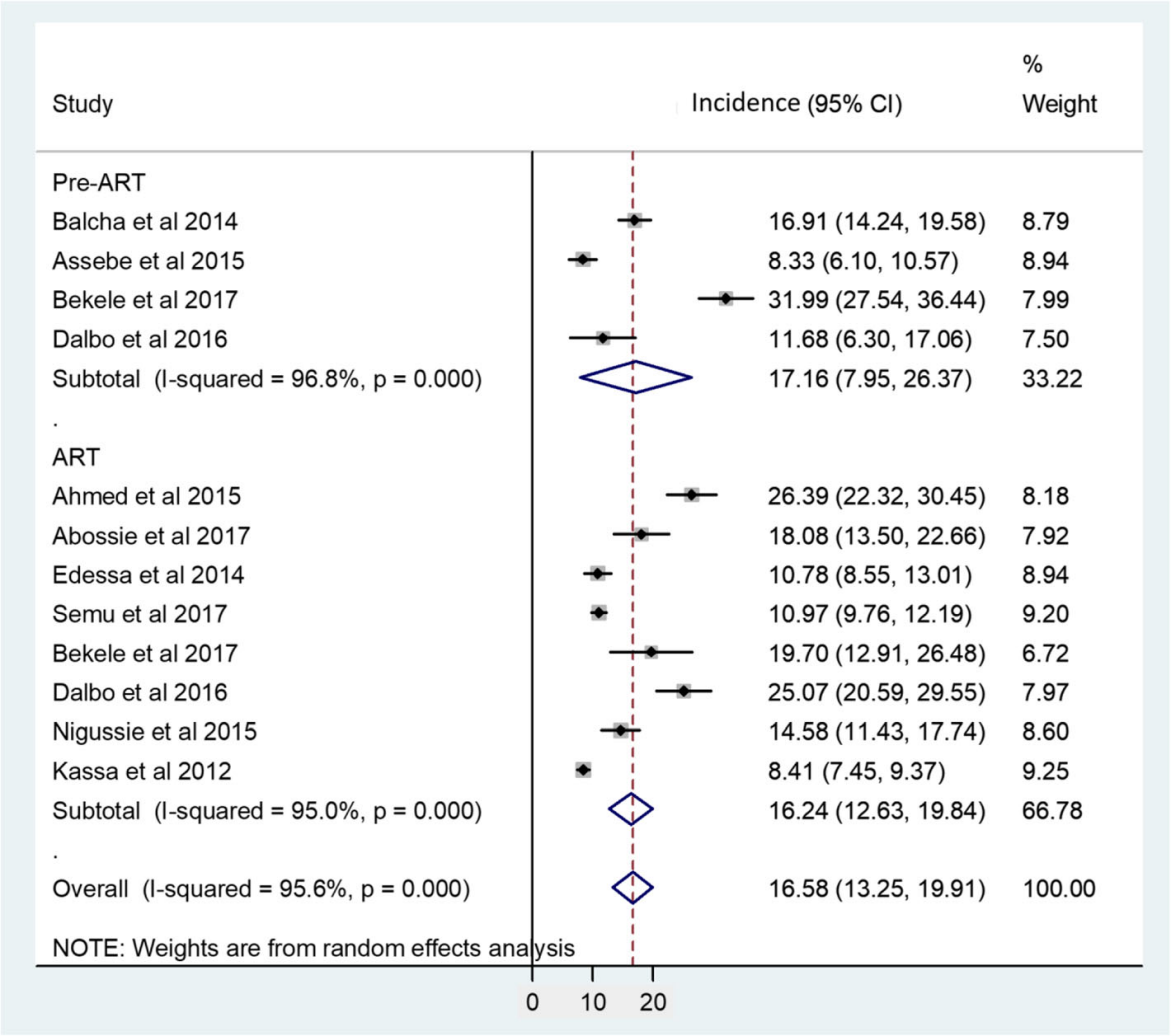
Forest plot representing pooled incident TB among adult HIV positive patient on pre-ART and ART in Ethiopia

The overall pooled incident TB among adult HIV positive patients in this meta-analysis from the random effects model was 16.58% (95% CI; 13.25–19.91%). Subgroup analysis based on patients ART status showed that 17.16% (95% CI; 7.95–26.37%) and 16.24% (95% CI; 12.63–19.84%) TB incidence on pre-ART and ART taking patients respectively (Fig. 3).

Moreover, subgroup analysis was done based on ART follow up time (6 months versus > 6 months) and baseline CD4^+^ T cell counts (< 200 versus > 200 cells/mm^3^). Accordingly, the pooled TB incidence among adult HIV infected patients on ART within 6 and >  6 months of follow up was 14.15% (95% CI; 7.01–21.28%: I^2^ = 87.3%) and 14.59% (95% CI; 10.06–19.13%: I^2^ = 96.4%) correspondingly. Likewise, the pooled incidence TB among adult HIV positive patients on ART with baseline CD4^+^ T cell count < 200 and > 200 cells/mm^3^ was 28.86% (95% CI; 18.73–38.98%: I^2^ = 88.5%) and 13.7% (95% CI; 1.41–25.98%: I^2^ = 95.8%) respectively (Table 3).

**Table 3:** Summary estimates for subgroup analysis of pooled TB incidence among included studies

Variables	Subgroups	Included studies	TB incidence (95% Cl)	I^2^
ART follow up time (months)	< 6 months	2	14.15% (7.01–21.28%)	87.3%
	> 6 months	4	14.59% (10.06–19.13%)	96.4%
Baseline CD4^+^ T cell count	< 200 cells/mm^3^	3	28.86% (18.73–38.98%)	88.5%
	> 200 cells/mm^3^	3	13.7% (1.41–25.98%)	95.8%

#### Association between CD4^+^ T cell count and the risk of incident TB

**Fig. 4:**
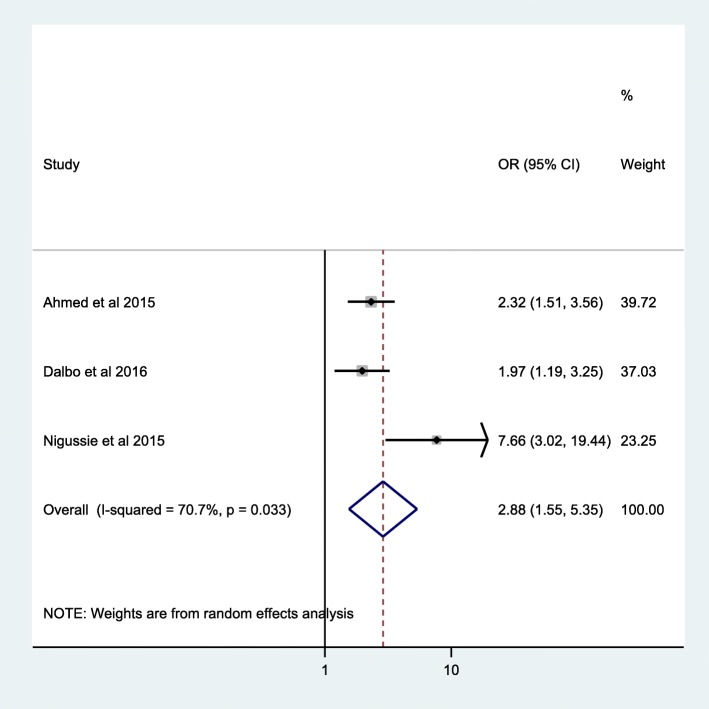
Forest plot of the pooled odds ratio showing the association of baseline CD4^+^ T cell count less than 200 cells/mm^3^ with incident TB among adult HIV positive patients on ART

Among 3 studies [27, 28, 30] reporting incident TB data in HIV positive patients on ART with both strata of baseline CD4^+^ T cell counts, we have determined incident TB among HIV infected patients with CD4^+^ T cell count < 200 cells/mm^3^ compared to HIV infected patients with CD4^+^ T cell count > 200 cells/mm^3^. Consequently, the odds ratio for incident TB among HIV positive patients with CD4^+^ T cell count < 200 cells/mm^3^ was 2.88 (95% CI; 1.55–5.35%: I^2^ = 70.7%: Egger’s test, *p* = 0.27) compared to HIV infected patients with CD4^+^ T cell count > 200 cells/mm^3^. In clinical perspectives, it means that HIV positive patients with CD4^+^ T cell count < 200 cells/mm^3^ have 2.88 times more likely to develop incident TB compared with patients who have CD4^+^ T cell count > 200 cells/mm^3^ (Fig. 4).

## Discussion

HIV fueled incident TB is resurging in developing countries, especially in sub-Saharan Africa where HIV prevalence is rampant. CD4^+^ T lymphocytes has a crucial role in both HIV and TB infection, and a decreased CD4^+^ T cell count has been implicated as a strong predictor of TB risk in HIV infected patients [31]. Thus, this meta-analysis was aimed to determine pooled incident TB and its association with baseline CD4^+^ T cell count among adult HIV infected patients in Ethiopia.

Based on this meta-analysis, the overall pooled incident TB was 16.58% (95% CI; 13.25–19.91%). This study demonstrated higher incident TB among adult HIV positive patients compared to the Ethiopian national population based TB prevalence survey, 261 per 100,000 person incident TB [32] among the general population of Ethiopia. This could be because of the difference in the study population. Our review summarized the finding in HIV positive patients who are at higher risk of developing TB [2] unlike the national population based survey that predominantly included HIV uninfected population. Partly, it may be due to national TB prevalence survey reports are rough estimates, and may underestimate the actual picture of TB incidence in the country.

As indicated in subgroup analysis, TB incidence in Pre-ART and ART taking patients was 17.16% (95% CI; 7.95–26.37%) and 16.24% (95% CI; 12.63–19.84%) respectively. However, previous studies revealed 21.63 and 14.27% in India [33], 7.9 and 4.4% in Tanzania [34] and 10.64 and 3.41% in South Africa [35] incident TB among pre-ART and ART receiving patients respectively. Therefore, except pre-ART incident TB reported in India, this study revealed higher incident TB report relative to other high TB burden settings, including South Africa [5, 36, 37]. This might be because of the clinical picture of the diseases may vary in different countries due to socio-economic or socio-cultural variations. Moreover, this study also demonstrated higher incident TB among HIV positive patients receiving ART in Ethiopia compared to previous studies established elsewhere. This sustained susceptibility to TB regardless of ART status in Ethiopia may be due to ongoing community level TB transmission, or possibly nosocomial TB transmission. As it is shown in this study, incident TB in pre-ART, 17.16% (95% CI; 7.95–26.37%), and ART, 16.24% (95% CI; 12.63–19.84%), was almost the same. This could be partially explained by the immune reconstitution inflammatory syndrome (IRIS), that can increase incident TB within the first one to 2 months following ART initiation among severe immunodeficiency patients [38], thereby mystifying the efficacy of ART to avert TB. On the other hand, it might be because of delayed HIV diagnosis and ART initiation, or due to poor ART adherence.

Further, the aggregate TB incidence among adult HIV positive patients within 6 and >  6 months of ART follow up was also determined as 14.15% (95% CI; 7.01–21.28%) and 14.59% (95% CI; 10.06–19.13%) respectively. This study suggests slightly increasing in TB incidence with increasing ART follow up time. However, previous studies indicated that incident TB was higher within the first 6 months of ART initiation than those who have been on ART for more than 6 months and longer [39]. This might be because of poor ART adherence and less concomitant use of TB preventive therapy in this study participants than previously established studies.

In addition, the pooled incident TB among adult HIV positive patients on ART with baseline CD4^+^ T cell counts < and > 200 cells/mm^3^ was also measured in this meta-analysis. Thus, this study revealed 28.86% (95% CI; 18.73–38.98%) and 13.7% (95% CI; 1.41–25.98%) incident TB among adult HIV infected patients on ART with baseline CD4^+^ T cell counts < and > 200 cells/mm^3^ respectively. Nevertheless, earlier studies demonstrated 1.90 and 1.52% in South Africa [35], 4.98 and 0.63% in Uganda [40], and 0.6 and 0.0% in Brazil [41] incident TB among HIV positive patients on ART with baseline CD4^+^ T cell counts < and > 200 cells/mm^3^ respectively. Thus, this study revealed higher incident TB compared to previous studies reporting incidence TB among HIV infected patients receiving ART with both baseline CD4^+^ T cell counts < 200 and/or > 200 cells/mm^3^. This might be due to higher TB transmission rate at the community level in Ethiopia compared to other study settings. Moreover, as ruling out TB in HIV positive patients on ART is challenging due to poor sensitivity of the sputum smear and also WHO symptom screening [42], the actual burden of incidence TB might be even higher than the stated one in our study. High incident TB was demonstrated in adult HIV infected patients on ART with baseline CD4^+^ T cell counts < 200 cells/mm^3^ relative to patients with CD4^+^ T cell counts > 200 cells/mm^3^ in this study. Consistent to our finding, previous meta-analysis study in South Africa indicated that the proportion of incident TB cases were increased as the baseline CD4^+^ T cell count decreased, especially when it was below 200 cells/mm^3^ [43]. There are enormous evidences that noted that baseline CD4^+^ T cell counts < 200 cells/mm^3^ is associated with increased incidence TB [8, 40, 41, 44]. This might happen for two reasons. First, this could be partly due to impaired restoration of TB specific immunity when patients are severely immunocompromised (baseline CD4^+^ T cell counts < 200 cells/mm^3^) at ART initiation [36]. Second, it might be because of primary infection or re-infection with the bacilli or re-activation of the existing latent TB as a result of severe immunosuppression.

Besides, the quantitative correspondence between baseline CD4^+^ T cell count and TB risk was measured in this meta-analysis. Consequently, adult HIV positive patients on ART with baseline CD4^+^ T cell count < 200 cells/mm^3^ were 2.88 times more likely to develop incident TB compared to patients with CD4^+^ T cell count > 200 cells/mm^3^. A comparable result was also reported in previous study from rural South Africa, three fold [36] and Tanzania, 5 to 20% higher risk of TB among HIV infected patients with CD4^+^ T cell count < 200 cells/mm^3^ [34]. There are a large number of evidences that showed baseline CD4^+^ T cell count < 200 cells/mm^3^ as a risk factor for development of incident TB in HIV positive patients [8, 36, 40, 41, 44]. This could be because of HIV induced depletion of CD4^+^ T lymphocytes leads to impaired cellular immunity and increased vulnerability to opportunistic infections like TB or reactivation of the latent TB [7] even in the presence of ART. Besides CD4^+^ T lymphocytes, HIV has also effects on antigen presenting cells like macrophages, and affects antigen processing and presentation as well cytokine production, which might also prevent the host from having an initial or latent TB infection [45]. Therefore, the result of this study indicates Ethiopian Federal Ministry of Health and HIV program managers in the country to increase the coverage of TB preventive therapy, adherence to ART and TB preventive therapy for HIV positive patients as per the recently recommended treatment guideline [46].

### Limitations of the study

Although the binomial distribution of meta-analysis was preferred to model within-study variability of the proportion [47], we used the usual DerSimonian and Laird random effects model to meta-analyze the proportion of incident TB. Repeated CD4^+^ T cell count measurement and the corresponding incident TB was not determined in this meta-analysis due to lack of report in included studies. CD4^+^ T cell counts and the risk of incident TB among pre-ART patients was not evaluated due to lack of CD4^+^ T cell count in the original studies. Besides, incident TB and duration of ART is not characterized in this study due to lack of clear evidence in the original studies. Although IRIS associated incident TB might be one of the factors for higher TB incidence among ART receiving patients, IRIS related incident TB was not documented in this study due to lack of information in the included studies. To report incidence rate, we were unable to find the details of each individual data, how many days/months/years spent each study participant in the observation. Therefore, in the absence of each participant’s detailed data in the observation, we reported incidence proportion rather than incidence rate in this study.

## Conclusions

This meta-analysis demonstrated that incident TB was considerably high in Ethiopia, especially in HIV positive patients with baseline CD4^+^ T cell count < 200 cells/mm^3^. Therefore, early HIV screening and ART initiation, as well as strict compliance with ART and increasing the coverage of TB preventive therapy to the more risky groups are important to overcome the problem in Ethiopia.

## Supplementary information

**Additional file 1.**

## Data Availability

All data pertaining to this study are contained and presented in this manuscript.

## Abbreviations

ART: Antiretroviral therapy
CD4: Cluster of differentiation 4;
CI: Confidence interval
FMH: Federal Ministry of Health
HIV: Human immunodeficiency virus
OR: Odds ratio
PLWH: People living with HIV
TB: Tuberculosis
WHO: World health organization.
