# Supplementary material for: Tuberculosis and its association with CD4+ T cell count among adult HIV positive patients in Ethiopian settings: a systematic review and meta-analysis
Source: BMC Infect Dis. 2020 May 7;20:325. doi: 10.1186/s12879-020-05040-4 (PMC7204319; doi:10.1186/s12879-020-05040-4)
Supplement: Supplementary file 1 — Additional file 1. [file 12879_2020_5040_MOESM1_ESM.docx]

**PubMed search string**

[("human immunodeficiency virus" OR "human immunodeficiency virus"[MeSH Terms] OR HIV OR HIV[MeSH Terms]) AND ("CD4+ T cell count" OR "CD4+ T cell count"[MeSH Terms]) OR “CD4 count”) OR “CD4 count”[MeSH Terms]) AND (Tuberculosis OR TB OR Tuberculosis[MeSH Terms] OR TB[MeSH Terms]) AND (infection OR incidence OR infection[MeSH Terms] OR incidence[MeSH Terms]) AND (Ethiopia OR Ethiopia[MeSH Terms])]
